# Polyarteritis Nodosa With Mesenteric, Hepatic, and Musculoskeletal Manifestations in a 36-Year-Old Nigerian Male Patient: A Case Report

**DOI:** 10.7759/cureus.97049

**Published:** 2025-11-17

**Authors:** Oluyemisi Okwudishu, Olasunkanmi Olatunbosun, Chika S Chijioke, Victor C Okebalama, Bonaventure Ogbodo, Olutomiwa Omokore, Joshua Odeyinka, Jennifer O Ibhiedu, Onyebuchukwu Eke, Obioma Aririsukwu

**Affiliations:** 1 Department of Internal Medicine, Babcock University Teaching Hospital, Ilishan-Remo, NGA; 2 Department of Acute Medicine, Wye Valley NHS, Hereford, GBR; 3 Department of Haematology, University Hospital of Wales, Cardiff, GBR; 4 Department of Anatomic Pathology and Forensic Medicine, Babcock University Teaching Hospital, Ilishan-Remo, NGA; 5 Department of Morbid Anatomy, University of Nigeria Teaching Hospital, Enugu, NGA; 6 Department of Internal Medicine, Benjamin S. Carson College of Health and Medical Sciences, Ilishan-Remo, NGA; 7 Department of Surgery, Babcock University Teaching Hospital, Ilishan-Remo, NGA; 8 Department of Pharmacology and Therapeutics, Ebonyi State University, Abakaliki, NGA; 9 Department of Medicine, St Francois Medical Centre, Abuja, NGA

**Keywords:** anca-negative vasculitis, immunosuppressive therapy, ischemic complications, nigerian male, polyarteritis nodosa, systemic vasculitis

## Abstract

Polyarteritis nodosa (PAN) is a rare systemic necrotizing vasculitis affecting medium and small arteries, with heterogeneous clinical manifestations ranging from constitutional symptoms to life-threatening organ ischemia. We present the case of a 36-year-old male patient with PAN manifesting as severe abdominal pain and bilateral upper limb ischemia, highlighting the diagnostic and therapeutic challenges of this condition. Initial evaluation revealed elevated inflammatory markers and characteristic arterial stenosis with aneurysmal dilation on Doppler ultrasound, supporting a PAN diagnosis with negative antineutrophil cytoplasmic antibody (ANCA) serology. Treatment with high-dose glucocorticoids induced remission, underscoring the efficacy of immunosuppression in managing this disease.

This case illustrates the importance of considering PAN in patients with unexplained ischemic symptoms, particularly in the absence of ANCA positivity. While glucocorticoids remain first-line therapy, emerging evidence supports the use of biologic agents in refractory cases. The discussion explores the evolving treatment paradigms, including the role of cyclophosphamide, tumor necrosis factor (TNF) inhibitors, and rituximab, while emphasizing the need for risk-stratified management. Given PAN’s variable presentation and potential for severe complications, early recognition and tailored immunosuppressive therapy are crucial for optimizing outcomes.

## Introduction

Polyarteritis nodosa (PAN) is a rare systemic necrotizing vasculitis of unknown etiology. PAN affects medium and small arteries all over the body, involving the skin, kidneys, central nervous system, and muscles, usually sparing the lungs [1]. 

PAN typically presents in the fifth decade of life, with its prevalence reducing due to the vaccination against hepatitis B virus (HBV), with up to 30% of PAN occurring with HBV before the inception of the vaccine [2,3].

The clinical findings of PAN are dependent on the parts of the body affected by the disease, with the affected organs showing areas of ischaemia on imaging. There is marked elevation of inflammatory markers without an infectious etiology, and angiography is remarkable for areas of arterial stenosis with intervening areas of aneurysmal dilation with or without arterial dissection. Biopsy of affected vessels is the gold standard of diagnosis, but it is deferred in most cases because of the risks associated with it [3].

Treatment of PAN is typically with high-dose steroids and oral cyclophosphamide. However, newer modalities with biological agents, including tumor necrosis factor (TNF) inhibitors (infliximab, adalimumab) and rituximab, have been used for refractory cases [4].

We present the case of a 36-year-old man who presented with features of systemic PAN affecting the gastrointestinal and musculoskeletal systems.

## Case presentation

A 36-year-old man presented to the emergency room with a sudden onset of abdominal pain. The pain started 24 hours before presentation, was periumbilical, and was described as dull aching. The intensity and severity worsened over time, necessitating his admission. Abdominal examination revealed severe tenderness in the periumbilical region.

The renal function test, urinalysis, and urine and blood culture were unremarkable (Table 1). His urine toxicology showed recreational use of THC and benzodiazepines. Viral markers are negative.

**Table 1:** A summary of patient’s investigation results

Investigations	Result	Reference Intervals
Inflammatory marker		
Erythrocyte sedimentation rate	>100mm/hr	
C-reactive protein	130	0-10 mg/l
Antibodies		
Centromere antibody	0.9	0-10 U/ml
Rheumatoid factor	8.21	0-20 IU/L
Liver function test		
Alanine transaminase	215	<40 IU/L
Aspartate transaminase	134	<40 IU/L
Gamma-glutamyl transaminase	520	7-50IU/L
Alkaline phosphatase	942	<225 IU/L
Total bilirubin	7.7	1.7-7.7 umol/L
Direct bilirubin	4.1	0-6.8 umol/L
Albumin	4.0	3.5-5.2 g/dL
Globulin	3.8	2-4 g/dL
Total protein	7.8	6-8 g/dL
Full blood count		
Total white cell count	9.4	4-11 (/L x 10^9)
Neutrophil (%)	77.3	50-70%
Lymphocyte (%)	9.1	20-30%
Monocyte (%)	10.7	3-12%
Eosinophil (%)	0.6	0.5-5%
Basophil (%)	2.3	0-1%
Red cell count	4.44	4-5-5.5 (X 10^12)
Haematocrit	13.0	40-54%
Platelet count	524	150-450 (/L x 10^9)
Renal function test		
Sodium	138	135-145 mmol/L
Potassium	3.6	3.5-5.0 mmol/L
Bicarbonate	21	20-31 mmol/L
Chloride	89	95-110 mmol/L
Urea	5.4	1.7-9.1 mmol/L
Creatinine	91	53-115 umol/L
Clotting profile		
Prothrombin time	16 seconds	17 seconds
International normalised ratio	1.3	0.8-1.2
Activated partial thromboplastin time	42 seconds	38 seconds

Abdominopelvic CT and ultrasound scans were unremarkable; abdominal X-ray showed multiple distended bowel loops with multiple air-fluid levels. An initial assessment of acute liver disease secondary to substance abuse and partial intestinal obstruction was made. However, abdominal pain subsided, and the patient had bowel movements and was subsequently discharged home.

He presented to the ER 48 hours after discharge with severe pain and swelling in both upper limbs; the pain was dull and aching, said to be a severity of 10/10, and it was aggravated by movement. Physical examination revealed severe tenderness and mild hyperpigmentation all over the bilateral upper limbs, with differential warmth with palpable peripheral pulses. The biopsy was deferred due to the high bleeding risk. Arterial and venous Doppler USS of both upper limbs showed severe bilateral ulnar artery stenosis with intervening areas of stenosis, with significant peripheral artery disease. The venous component of the study was unremarkable. Echo was normal. Rheumatoid factor and ANCA were negative. Differential diagnoses, including thromboangiitis obliterans, cannabis arteritis, and HBV-related vasculitis, were all excluded based on the presentation and investigation findings.

An assessment of PAN was made, and the patient was referred to rheumatology. He was placed on a high-dose steroid (oral prednisone 70 mg daily) as the induction phase of his treatment for a month. Cyclophosphamide was not considered, as he responded well to the steroid. He was later commenced on the maintenance dose of oral prednisone, which was tapered down to 10 mg daily, and has been symptom-free for more than one year now.

## Discussion

PAN is a systemic inflammation of medium and small arteries, sparing arterioles, venules, and capillaries. It can affect patients of all ethnic backgrounds and genders with a predilection for the male sex (1.5:1). PAN has a prevalence of around 33 per million and an incidence of 0-1.6 cases per million per year [5]. PAN can affect all age groups, with its highest incidence in the fifth and sixth decades [5]. Our patient is a 36-year-old male of African heritage.

There are multiple etiologies associated with PAN reported in the literature, including infective, hematological, and autoimmune conditions. It could also be idiopathic. Historically, 30% of PAN was associated with HBV infection. The prevalence has decreased significantly because the hepatitis vaccine is now routine in many countries worldwide [2-4].

The clinical presentation of PAN is heterogeneous and depends on the part of the body affected. Patients present with nonspecific constitutional symptoms like asthenia, fever, arthralgia, myalgia, and weight loss. PAN can affect any organ in the body (usually sparing the lungs). It has a predilection for the integumentary, nervous, and musculoskeletal systems. The lack of granulomas, glomerulonephritis, and ANCAs helps differentiate PAN from other forms of vasculitis. The disease is characterized by major thrombotic and hemorrhagic complications, particularly affecting the renal, mesenteric, cerebral, and cardiac systems [3,6]. Our patient had severe poorly localized abdominal pain with elevated liver enzymes and severe bilateral upper limb pain, which was suggestive of hepatic and mesenteric (particularly the liver and intestines), a rare manifestation of PAN with associated increased morbidity and mortality [3] and musculoskeletal thrombotic and ischemic events.

Smoking in general is associated with ANCA-associated vasculitis, and the use of cannabis is associated with cannabis arteritis [7,8]. Cannabis arteritis is a diagnosis of exclusion with a similar presentation to thromboangiitis obliterans; some authors consider it a subtype of thromboangiitis obliterans because they have the same clinical features and appearance on arteriography [8]. Although our patient used cannabis, the clinical presentation of abdominal pain, upper limb pain, and the presence of a dilated artery with intervening areas of stenosis on Doppler interrogation of both upper limbs, with a negative ANCA test, made a diagnosis of PAN more likely.

The laboratory work-up of PAN is peculiar to the organs affected. However, ANCA negativity and a characteristic pattern seen on angiography, where multiple aneurysms and constrictions along an affected artery resemble beads on a string, are strong indicators for PAN. Although histology is the final arbiter for the diagnosis of PAN, it is rarely done because of the risk of hemorrhage or thrombosis. Histology typically shows transmural fibrinoid necrosis of the arterial wall with infiltration of polymorphonuclear cells [3,5]. In our case, the benefit of taking a biopsy was overshadowed by the risk of hemorrhage of mesenteric vasculature; the skin biopsy was normal, as cutaneous biopsies show vasculitis in approximately 50% of patients [5].

PAN presents across a spectrum of severity, necessitating tailored therapeutic strategies. For milder cases characterized by non-life-threatening manifestations such as constitutional symptoms, arthritis, or cutaneous involvement, treatment typically centers on glucocorticoid monotherapy. However, emerging evidence suggests that supplementing corticosteroids with immunosuppressive agents like azathioprine or methotrexate may offer advantages in reducing the substantial 40% relapse rate and minimizing steroid-related complications. This approach reflects evolving treatment paradigms, with current guidelines demonstrating some divergence, while French protocols favor initial glucocorticoid monotherapy, reserving immunosuppressants for refractory cases. The ACR's 2021 recommendations advocate for earlier combination therapy [9,10].

More severe disease presentations, marked by critical organ involvement such as renal artery stenosis, cardiomyopathy, or gastrointestinal ischemia, demand aggressive intervention. The cornerstone of treatment involves high-dose intravenous glucocorticoids combined with cyclophosphamide during the induction phase, typically spanning three to six months. A prospective trial showed a significantly reduced relapse probability (P = 0.02; hazard ratio (HR) = 0.34) and increased event-free survival (P = 0.02, HR = 0.44) in patients who received the combination of the aforementioned drugs [11]. Furthermore, particularly fulminant cases may benefit from methylprednisolone pulse therapy. Following successful remission induction, patients transition to maintenance therapy with alternative immunosuppressants, continuing for 12 to 18 months to prevent disease recurrence.

The management of refractory PAN presents ongoing challenges, with various biological agents, including rituximab, TNF-α inhibitors, and tocilizumab, demonstrating variable efficacy in small studies. Recent European data indicate response rates approaching 50% for some targeted therapies, though their precise role requires further clarification through controlled trials [6]. Our patient had high-dose corticosteroid therapy and has been symptom-free.

## Conclusions

In conclusion, PAN remains a complex and potentially life-threatening vasculitis requiring a high index of suspicion for diagnosis, particularly in patients presenting with unexplained ischemia or systemic inflammation. This case highlights the diagnostic utility of angiography and the challenges of histologic confirmation due to procedural risks. Glucocorticoids, often combined with cyclophosphamide, remain the cornerstone of therapy; however, emerging biologic agents show promise for treating refractory disease. The management of PAN continues to evolve, with current guidelines advocating for risk-adapted strategies-glucocorticoid monotherapy for mild cases and aggressive immunosuppression for severe or organ-threatening disease. Future research should focus on refining diagnostic biomarkers and optimizing biologic therapies to improve long-term outcomes. Clinicians must remain vigilant for atypical presentations, as early intervention significantly reduces morbidity and mortality in this rare but devastating condition.
